# Training Programs in Communication Skills for Health Care Professionals and Volunteers

**DOI:** 10.4103/0973-1075.76232

**Published:** 2011-01

**Authors:** KC Rajashree

**Affiliations:** Senior Registrar, Pain and Palliative Care Clinic, Kozhikode, Kerala, India

**Keywords:** Communication skills, Training programmes, Palliative care

## Abstract

Communication skills are as important as vital needs. Health care professionals have to be aware of their own communication practices and need to undergo periodic appraisal of the same. Training programmes in communication skills are unfortunately not part of our academic curriculum. The article highlights the need and the overview of such training programmes.

## INTRODUCTION

Patient–professional communication is a fundamental skill of medical practice. It is the building block on which a therapeutic relationship with the patient is constructed. By understanding a patient in a holistic manner, the doctor health care provider can draw a total care plan which is best suited for that patient.

Communication is a two way process which has both verbal as well as nonverbal components. Most of us believe that we communicate more through verbal means but it is not so. Concentrating more on the verbal content and ignoring the nonverbal means can make communication less effective. A seemingly straightforward communication may become quite challenging due to the complexity of the whole processes involved. This happens because the interaction between a health care professional and a patient are influenced by the feelings (emotions) and thoughts by both the parties which are at different levels surrounded by the social context and the environment where the communication takes place. On the other hand, a good communication can improve patient outcome, patient and physician satisfaction.

## PRESENT SCENARIO

The academic curriculum for medical students lacks any training methods on improving communication skills. The emphasis is not given during the clinical discussion also. While it is simple to incorporate small sessions on communication skills in the routine teaching schedule, it is unfortunate to say that hardly any attempt is made.

## OVERVIEW OF THE TRAINING PROGRAMS

The student (MBBS/BDS/Nursing) in the first year of his or her training period learns the basic sciences and then moves to the clinical subjects. This the best period to start the training programs as they can practice the principles of communication when they see the patients in the inpatients and outpatient units.

The preliminary training should focus on the basics of communication. This should cover

- the general introduction and the need of proper communication;
- common difficulties encountered by the professionals and patients in a clinical setting. This throws light into a totally neglected idea of patient’s perspectives of having an illness and helps the student to identify the patient as a person with a disease rather than a “case”;
- listening techniques which encourage the patients to talk more and facilitate the interaction between the patient and the professional;
- common barriers which occur in our communication practice. This will enable the student to understand that we often express distancing behavior unknowingly, which can strain the relationship with the patient.

These topics can be covered in a three-hour session. To make the session more interactive, role plays and group discussion can be included. We can also do a pretest evaluation to assess the participant’s understanding about communication and a post-test evaluation with the same questionnaire to assess the change in knowledge as well as the attitude.

We need a detailed training program for the practicing health care professionals regarding more delicate and complex issues like breaking bad news and handling various reactions by the patients and the relatives. This necessitates the skill of the trainer in facilitating the discussion as most of the health care professionals hold a strong conviction about these issues. The session should include previous topics mentioned for the students and issues in breaking bad news and handling reactions. These sessions are handled through fewer lectures and more role plays which highlight common issues that can occur in a professional patient interaction and discussion based on the role plays. The training session needs at least six hours for an effective interaction.

In both the sessions (student and practicing professionals) use of videos acted by mock patients and the trainer or clippings from movies can be included which will make the session more effective and interesting.

We can conduct the similar training programs for volunteers also. They need more emphasis on the listening techniques and the common barriers. The preliminary training can be completed in a three-hour session and a detailed session is planned after they go for home visit. This will enable the volunteer to identify and understand the issues in a chronically ill patient in a better way and is an integral part of the volunteers’ training programs run in Kerala which has 16 hour duration. It is done in the regional language.

For volunteers we also conduct an advanced training program. This is of 100 hour duration and the session is done in four phases of 25 hours duration each. It covers the previously mentioned topics for the preliminary training and issues in breaking bad news and handling reactions. The session is handled through extensive number of role pays, group work, assignment discussion, etc. The sessions are mainly facilitated by the group of volunteers who have immense experience in patient inter action and handling training sessions. This advanced training program has become very successful and we have already completed two batches of volunteers, each batch with approximately 50 volunteers.

## CONCLUSION

Good communication skills are essential for high quality, effective, and safe medical practice. These skills are used for information gathering, diagnosis, treatment, and patient education. Communication skills can be effectively trained but are best achieved through reviewing our own style of communication.

